# Supplementary material for: Clostridium difficile infection after stoma reversal surgery: a systematic review and meta-analysis of the literature
Source: Int J Colorectal Dis. 2024 May 29;39(1):81. doi: 10.1007/s00384-024-04643-6 (PMC11136761; doi:10.1007/s00384-024-04643-6)
Supplement: Supplementary file 1 — Supplementary file1 (DOCX 1350 kb) [file 384_2024_4643_MOESM1_ESM.docx]

***Clostridium difficile* infection after stoma reversal surgery: a systematic review and meta-analysis of the literature**

Tirelli Flavio^1^, Langellotti Lodovica^2^, Lorenzon Laura^1^, Biondi Alberto^1^, Santoro Gloria^1^, Pezzuto Roberto^1^, Agnes Annamaria^1^, D’Ugo Domenico^1^, Sanguinetti Maurizio^1^, Persiani Roberto^1^

^1^ Fondazione Policlinico Universitario Agostino Gemelli IRCCS, Catholic University of the Sacred Hearth, Rome, Italy

^2^ Catholic University of the Sacred Hearth, Rome, Italy

**Running Head.** *Clostridium difficile* infection and colorectal surgery

**Conflict of interest.** None of the authors has any relevant potential financial conflicts of interest related to this study.

**Data availability.** The data that support the findings of this study are not openly available to protect study participant privacy but are available from the corresponding author upon reasonable request.

**Article type.** Systematic review and meta-analysis

**Supplementary Materials Index**

| **Supplementary Methods** |  |
| --- | --- |
| PRISMA Check-List | *page 2* |
| **Supplementary Results** |  |
| Supplementary Figure 1 | *page 3* |

**Preferred Reporting Items for Systematic Reviews and Meta-Analyses (PRISMA) CHECK-LIST**

**
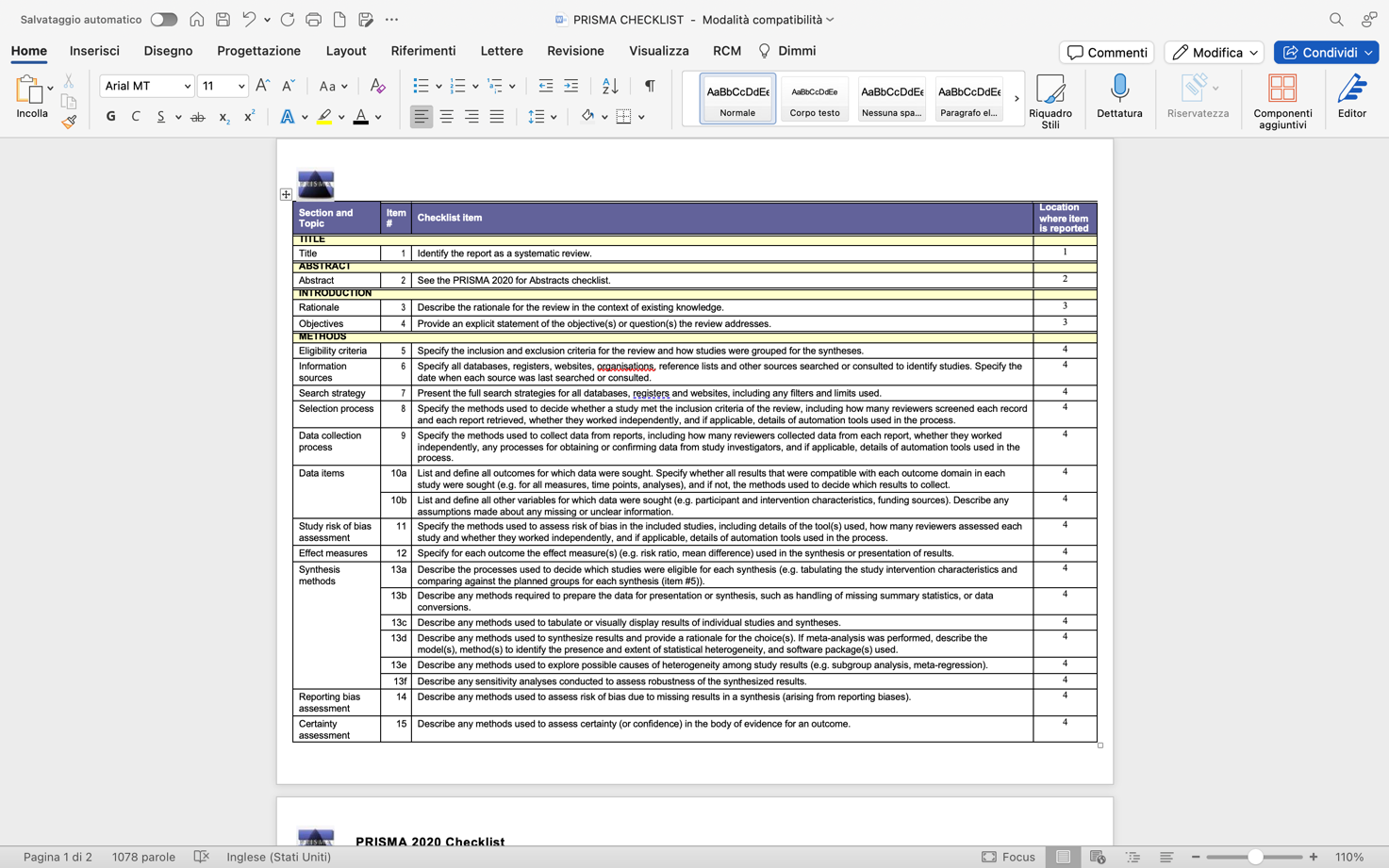
**


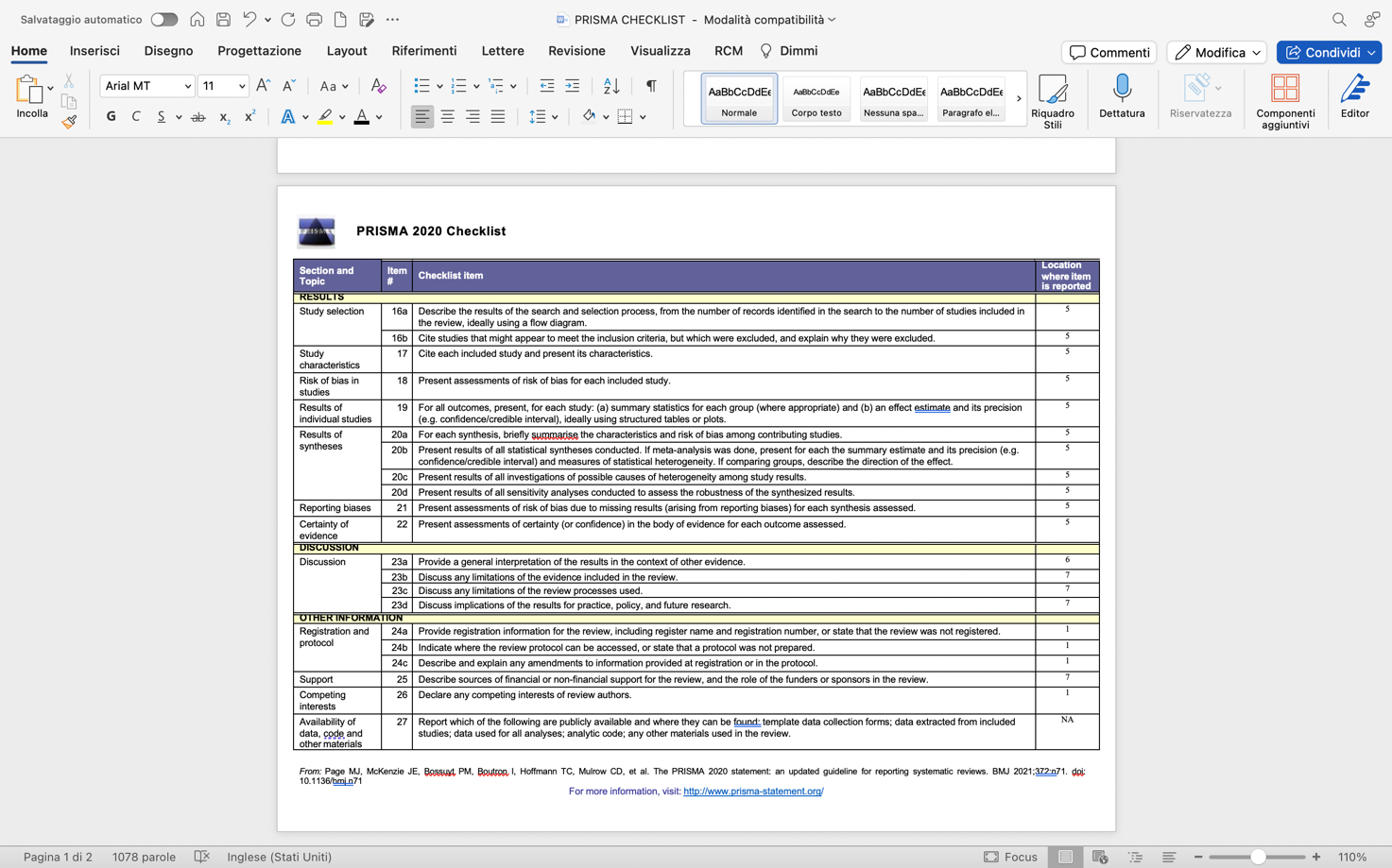


**Supplementary Figure 1:** Meta-analysis of proportion for CDI Infection.

1. **Forrest Plot B.** **Funnel Plot**

**
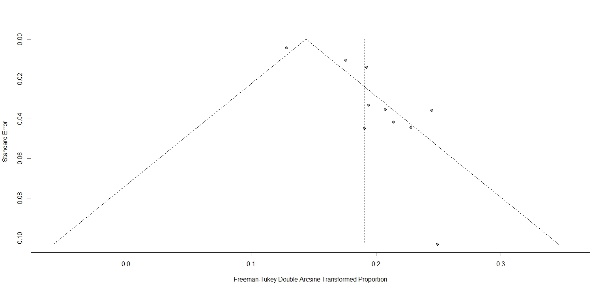

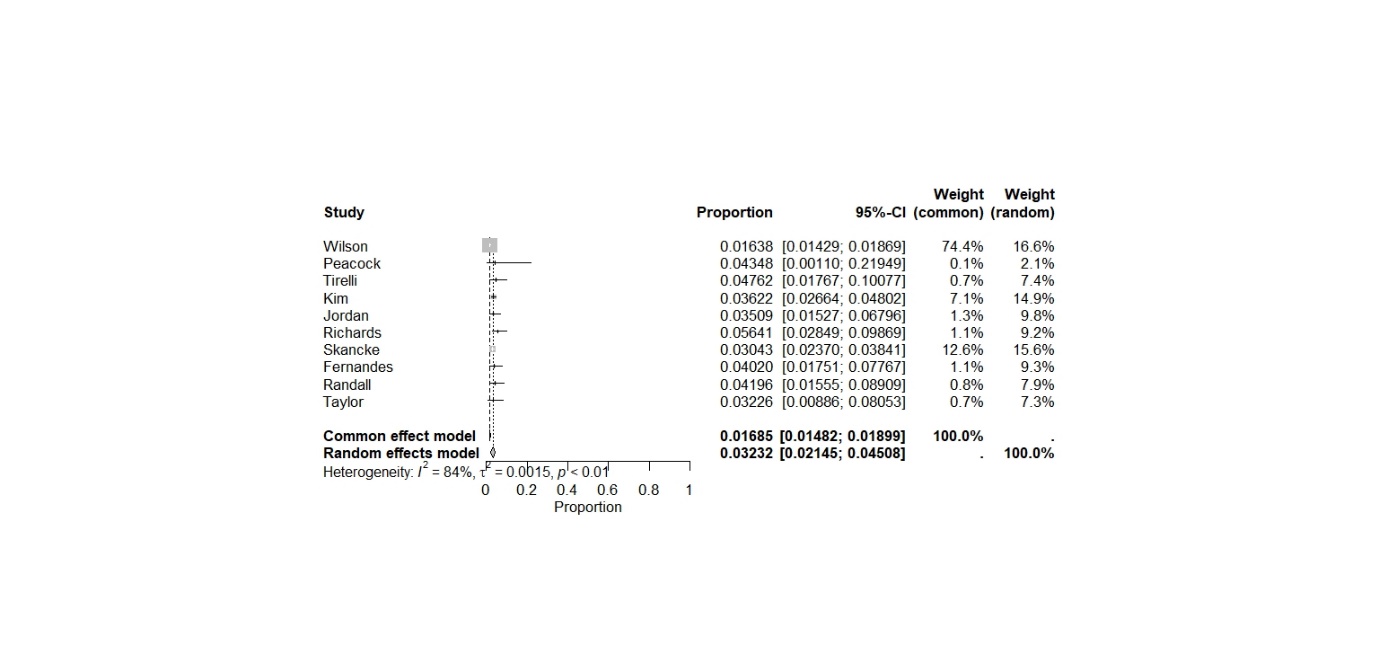
**
