# Supplementary material for: Clostridium difficile infection after stoma reversal surgery: a systematic review and meta-analysis of the literature
Source: Int J Colorectal Dis. 2024 May 29;39(1):81. doi: 10.1007/s00384-024-04643-6 (PMC11136761; doi:10.1007/s00384-024-04643-6)
Supplement: Supplementary file 5 — Supplementary file5 (PDF 1350 kb) [file 384_2024_4643_MOESM5_ESM.pdf]

Clostridium difficile infection after stoma reversal surgery: a systematic review and meta-analysis of the literature

Tirelli Flavio[1], Langellotti Lodovica[2], Lorenzon Laura[1], Biondi Alberto[1], Santoro Gloria[1], Pezzuto Roberto[1], Agnes Annamaria[1], D'Ugo Domenico[1], Sanguinetti Maurizio[1], Persiani Roberto[1]

[1] Fondazione Policlinico Universitario Agostino Gemelli IRCCS, Catholic University of the Sacred Hearth, Rome, Italy

[2] Catholic University of the Sacred Hearth, Rome, Italy

Corresponding author. Laura Lorenzon, Fondazione Policlinico Universitario A. Gemelli, Catholic University of the Sacred Hearth, Largo Francesco Vito 1, 00168 Rome, Italy. e-mail: laura.lorenzon@policlinicogemelli.it; phone number: +39-0630154974; fax +39-0630158015.

Running Head. *Clostridium difficile* infection and colorectal surgery

Conflict of interest. None of the authors has any relevant potential financial conflicts of interest related to this study.

Data availability. The data that support the findings of this study are not openly available to protect study participant privacy but are available from the corresponding author upon reasonable request.

Article type. Systematic review and meta-analysis

Word Count. Abstract: 298; **Manuscript**: 2253; **Figures**: 4; **Tables**: 2; **Supplementary Figure** 1.

PROSPERO registration number: CRD42023484704

Highlights.

- CDI occurs in approximately 2.1% of patients who undergo stoma reversal.
- A correlation with delayed stoma reversal and chemotherapy were observed in the CDI population.
- The meta-analysis documented a significant correlation between adjuvant therapy and CDI.

ABSTRACT

Background. *Clostridium difficile* infection (CDI) has been described in the early post-operative phase after stoma reversal. This systematic review aimed to describe the incidence of CDI after stoma reversal and to identify pre-operative variables correlated with an increased risk of infection.

Methods. A systematic review of the literature was conducted according to the PRISMA guidelines in March 2024. Manuscripts were included if reported at least one patient with CDI-associated diarrhoea following stoma reversal (colostomy/ileostomy). The primary outcome of interest was the incidence of CDI; the secondary outcome was the comparison of clinical variables (age, sex, time to stoma reversal, neo-adjuvant and adjuvant therapies after index CRC procedure) in CDI-positive versus CDI-negative patients. A meta-analysis was performed when at least 3 studies reported on those variables.

Results. Out of 43 eligible manuscripts, 1 RCT and 10 retrospective studies were selected, including 17857 patients (2.1% CDI). Overall, the mean age was 64.3±11.6 years in the CDI group and 61.5±12.6 years in the CDI-negative group (p= 0.51), with no significant difference in sex (p= 0.34). Univariable analyses documented that the mean time to stoma reversal was 53.9±19.1 weeks in CDI patients and 39.8±15.0 weeks in CDI-negative patients (p= 0.40), and a correlation between neo-adjuvant and adjuvant treatments with CDI (p<0.001). A meta-analysis was performed for time to stoma reversal, age, sex and neo-adjuvant therapies disclosing no significant differences for CDI (stoma delay: MD-11.59; 95%CI-24.32-1.13; age: MD-0.97; 95%CI-2.08-4.03; sex: OR-1.11; 95%CI-0.88-1.41; neo-adjuvant: OR-0.81; 95%CI-0.49-1.35). Meta-analysis including patients who underwent adjuvant therapy evidenced a higher risk of CDI (OR-2.88; 95%CI-1.01-8.17 p=0.11).

Conclusion. CDI occurs in approximately 2.1% of patients after stoma reversal. Although a trend of increased delay in stoma reversal and a correlation with chemotherapy were documented in CDI-patients, the use of adjuvant therapy was the only possible risk factor on meta-analysis.

Keywords. *Clostridium difficile* infection; stoma reversal; colostomy; ileostomy; colorectal surgery.

INTRODUCTION

Colorectal surgery has been identified as a risk factor for *Clostridium difficile* infection (CDI) [1-3], although its occurrence is relatively rare, since as it has been reported in only 1.5% of patients [4]. However, stoma closure surgery has the highest incidence rate of CDI among abdominal procedures, ranging from 1.6 to 8.7% of cases [5-14,21].

The creation of a diverting ileostomy or colostomy disrupts the normal anatomical structure of the bowel, leading to significant changes in the mucosal and muscular layers. This process also results in progressive atrophy of the dysfunctional colonic tract. The atrophy and decreased immune capacity of the colon mucosa increase its susceptibility to *Clostridium difficile* infection. [15]. Of note, Clostridium Difficile (CD) not only is a healthcare-related pathogen but also colonizes the gastrointestinal tract in 15% of the population. Usually, CD colonization is completely asymptomatic, but it can become symptomatic if normal intestinal flora disruption occurs [1-3].

The intestinal flora ecosystem plays a critical role in preserving the intestines by resisting colonization and infection by pathogenic organisms. Under normal circumstances, the human gut microbiota can hinder pathogen colonization through general mechanisms such as direct inhibition via bacteriocins, nutrient depletion, or stimulation of host immune defences. However, the specific mechanism by which the microbiota protects against CDI remains unknown. Disruption of the normal balance of the colonic microbiota can result from prolonged antimicrobial therapies, mucosal atrophy due to colonic faecal diversion, or immunosuppression [1-4].

There are several reasons why the incidence of CDI after stoma closure is greater than that after other colorectal procedures [16], including the clinical variables and intrinsic risk factors of patients undergoing stoma reversal surgery (elderly people with colon cancer, subjected to chemotherapy and radiotherapy, sometimes malnourished, or long-lasting stoma holders). [5]

We thus aimed this manuscript to systematically review the literature and describe the incidence of CDI after stoma reversal; the secondary outcome of interest was the comparison of the clinical variables in CDI-positive patients and CDI-negative patients.

METHODS

Data sources, search strategy and selection criteria. A systematic review of published papers was conducted according to the Preferred Reporting Items for Systematic Reviews and Meta-Analyses (PRISMA) guidelines in March 2024 (Supplementary Material). The following sources were searched for papers reporting cases of documented CDI following reversal of colostomy or ileostomy: PubMed, Embase and Medline. The search terms included (clostridium) AND (colorectal cancer), (clostridium) AND (colorectal surgery), (clostridium) AND (rectal cancer), (clostridium) AND (rectal surgery), (clostridium) AND (colostomy), (clostridium) AND (ileostomy), (clostridium) AND (stoma) (clostridium) AND (stoma closure), (clostridium) AND (stoma reversal), (clostridium) AND (stoma surgery) in all fields. The references of the included articles were also manually searched, and further articles were included if appropriate. Both ileostomy and colostomy reversal were considered for inclusion. The selection criteria included "English" languages, human studies, clinical trials, and observational and comparative studies. Duplicate references were semi-automatically removed using the RAYYAN platform (https://www.rayyan.ai/). Case reports were excluded. Each paper retrieved was assessed for inclusion or exclusion by revision of titles and abstracts by two authors (LL and FT), and any issues or disagreements were resolved by consensus.

Study risk-of-bias assessment. Randomized controlled trials (RCTs) were evaluated using the Robin 2.0 tool, whereas nonrandomized retrospective cohorts were evaluated using the Newcastle–Ottawa scale. GRADE criteria were considered to summarize evidences.

Outcomes of interest. The primary outcome of interest was the rate of CDI infection. The secondary outcome was to determine the association between clinical variables (age, sex, time to stoma closure, neo-adjuvant and adjuvant therapies) and CDI infection following stoma reversal in CDI-positive vs CDI-negative patients.

Statistics. Categorical variables were analysed using frequencies and percentages, and subgroups were compared using chi-square tests. Continuous variables were presented using means, standard deviations (SDs), medians, and ranges (IRQ). Meta-analyses were conducted when at least three studies provided computable variables. The Mantel-Haenszel method was used for calculating the weighted summary Odds Ratio (OR) under the fixed effects model. Next, the heterogeneity statistic is incorporated to calculate the summary odds ratio under the random effects model. The total odds ratio with 95%CI is given both for the Fixed effects model and the Random effects model. If the value 1 is not within the 95%CI, then the Odds ratio is statistically significant at the 5% level ($P < 0.05$). For meta-analysis of studies with a continuous measure (comparison of means between treated cases and controls), the Hedges g statistic was used as a formulation for the Mean difference (MD) under the fixed effects model. Next, the heterogeneity statistic is incorporated to calculate the summary standardized mean difference under the random effects model. If the value 0 is not within the 95%CI, then the MD is statistically significant at the 5% level ($P < 0.05$). Statistical heterogeneity

of the results of the papers was assessed on the basis of a test of heterogeneity (standard chi-squared test on N degrees of freedom where N equals the number of trials contributing data minus one). Three possible causes for heterogeneity were pre-specified: (1) differing response according to difference in the quality of the trial; (2) differing response according to sample size; and (3) differing response according to clinical heterogeneity. If the test of heterogeneity is statistically significant ($P < 0.05$) then more emphasis should be placed on the random effects model.

Furthermore, a meta-analysis of proportions was considered related to CD infection and total number of patients estimating the proportion of CDI in each study with the inverse of the variance weight, a measure of the precision of a weighted mean estimate; then it was calculated the standard deviation with the proportion estimate setting using the DerSimonian-Laird method, taking into account the variation between studies.

Statistical analysis was performed using R open-source software and the "*meta*" package in R (https://cran.r-project.org/). All tests were two-tailed, and p<0.05 was considered to indicate statistical significance.

RESULTS

Study population and systematic review. Out of a total of 1263 papers screened, 11 (10 retrospective cohort studies and 1 randomized controlled trial [5-14,21]) met the inclusion criteria and were analysed (Figure 1), including a previous paper from our group [5].

Table 1 shows the eleven papers analysed, including 17857 patients who underwent stoma reversal surgery (both ileostomy and colostomy reversal were considered for inclusion); the overall incidence of CDI was 2.1% (381/17857). [5-14,21]

Overall, the mean age of the CDI-positive patients was 64.3±11.6 years, while that of the CDI-negative patients was 61.5±12.6 years (p= 0.51), whereas the M/F ratio was 1.4 for the CDI-positive patients and 1.2 for the CDI-negative patients (p= 0.34) (Table 2). Additionally, the mean time to stoma reversal after the index CRC procedure was 53.9±19.1 weeks in CDI-positive patients and 39.8±15.0 weeks in CDI-negative patients (p= 0.40) (Table 2). Also, on univariable analysis, both neo-adjuvant and adjuvant therapy correlated with CDI: in particular, 11.8% of the patients who were treated with neo-adjuvant therapy developed a CDI vs 6.2% of those who did not, and 16.7% of patients who underwent adjuvant treatment had a CDI vs 6.5% of patients not treated with adjuvant chemotherapy, Table 2.

Study risk-of-bias assessment. There was one RCT [7] and ten non-randomized retrospective cohort studies [5,6,8-14,21]. All non-randomized studies scored 7 or more on the Newcastle–Ottawa scale, and the RCTs had a low risk of bias according to Robin 2.0. All studies were therefore deemed good quality studies (Figure 2).

Meta-analysis. Overall, five variables were computable for meta-analysis: age, sex, stoma closure delay, neo-adjuvant therapies, and adjuvant therapies in CDI-positive vs CDI-negative patients. The studies included in the meta-analyses [5,8-10] were based on 1591 patients for the time to stoma reversal variable, 1624 patients for the age variable, on patients 15064 for sex variable, on 549 patients for neo-adjuvant and adjuvant therapies. A meta-analysis of proportion documented that CDI infection rate was homogeneous among studies (Supplementary Materials, Supplementary Figure 1).

Figure 3 shows the meta-analysis on the delay of stoma closure and the meta-analysis on the mean age of patients and sex, documenting no significant differences between the CDI-positive and CDI-negative groups (stoma delay: MD-11.59; 95%CI-24.32-1.13; age: MD-0.97; 95%CI-2.08-4.03; sex: OR-1.11; 95%CI-0.88-1.41).

Figure 4 shows the meta-analysis for patients undergoing neo-adjuvant therapy (Figure 4-A) and documented no significant differences between the CDI-positive and CDI-negative groups (OR-0.81; 95%CI-0.49-1.35) whereas adjuvant therapies (Figure 4-B) showed a significative correlation between with the development of CD infection (OR-2.88; 95%CI-1.01-8.17 p=0.11).

DISCUSSION

This study analysed eleven papers including cases of *Clostridium difficile* infection after stoma reversal surgery. *Clostridium difficile* infection is an uncommon complication after stoma reversal surgery that occurs, according to this systematic review, approximately in 2.1% of the patients. Stoma closure surgery is an abdominal procedure with a relatively high incidence rate of CDI, ranging between 1.6 and 8.7% [5-14,21]. Although it is not a frequent complication, CDI is a potentially life-threatening complication, and it prolongs the costs and length of hospitalization.

Our group recently conducted a retrospective cohort study in a population of patients who underwent stoma reversal surgery after transanal total mesorectal excision (TaTME) for rectal cancer [5]. This surgical procedure has been adopted in our surgical unit since 2015, and it has become the treatment of choice for mid- and low rectal cancer, achieving successful surgical and functional outcomes [17]. The higher incidence of CDI in the postoperative period reported in this cohort (4.8%) could be explained by the characteristics of the study population (rectal cancers who underwent neo-adjuvant therapy in 72.2% of the cases and adjuvant therapy in nearly 48.4%), with a consequent delay in stoma closure. In this preliminary experience, we documented that delayed stoma closure was the main variable correlated with CDI; furthermore, the probability of presenting with diarrhoea symptoms was greater for males, increased with the comorbidity index, a lower mean albumin value, increased stoma delay closure, and the use of neo-adjuvant and adjuvant treatments.

Similarly, Jordan *et al.* [11] reported a 3.5% incidence of CDI (RR = 4.23). In their analysis, the CDI-positive group had a significantly longer median time to reversal, but this difference was not found to be statistically significant.

Indeed, most patients who undergo stoma closure surgery are previously treated with chemotherapies or radiotherapies that are toxic to the gut mucosa. In addition, it should be considered to the long-lasting defunctioning time, in cases requiring anti-neoplastic therapies or for organizational issues (i.e. Covid 19 pandemic).

The pathogenic mechanism of CDI in a defunctioning colon was explained by Kissmeyer-Nielsen [5] in 1994 through an experimental study on mice. Interruption of normal anatomical continuity by diverting ileostomy or colostomy causes radical morphological changes related to the mucosa and the muscular layers of the bowel. The composition of the colon wall in rats significantly changes following mucosal and muscular atrophy, as does the luminal surface area. Consequently, this alteration disrupts the normal balance of the colonic microbiota. In the context of dysbiosis, *Clostridium difficile*, which typically colonizes asymptomatic

individuals, becomes a hazardous pathogen. However, there are several micro-organisms that have the potential to become harmful to the gastrointestinal mucosa [18-19].

Although the use of antibiotics is an established risk factor for CDI [1], there are no clinical studies and sufficient data to correlate the use of a specific class of antibiotics in surgical prophylaxis with an increased incidence of CDI after stoma reversal surgery. Fernandez *et al.* [12] wrote the first paper demonstrating metronidazole as an effective preventative agent against postoperative diarrhoea and CDI in patients undergoing ileostomy reversal surgery. The results of this study indicated that single-dose metronidazole is more effective at reducing postoperative diarrhoea and CDI than multiple doses of cefuroxime plus metronidazole. Therefore, metronidazole may offer effective prophylaxis against CDI by reducing the CD load in the colon, permitting recolonization with indigenous gut microbiota. The preliminary results from this study need to be confirmed in large randomized controlled trials.

An interesting aspect for future studies could be to perform a preliminary CD test before surgery, in order to treat patients with a positive test to an adequate pre- and post-operative antibiotic therapy.

There may be some potential limitations in the present systematic review. The effects estimated in the model are based mainly on retrospective observational studies. They are therefore subjected to biases and confounding factors that may have influenced our model estimates. The main limitation of the included studies is the heterogeneity of the population. Heterogeneity in studies has been reported for a variety of reasons, including differences in the sample population regarding age, sex, BMI, and disease severity. Another limitation is the different study designs of the included papers (ten observational studies and 1 randomized clinical trial); otherwise, only observational studies were included in the meta-analysis. Other limitations are the low proportion of patients with CDI (limits statistical power when performing comparative tests), and lack of data pertaining to treatment and outcomes following CDI in these patients. Eventually, both ileostomy and colostomy are included in the same pool, and this is a difficult condition to adjust due to the lack of specific data in the included studies. Finally, a more extended literature search could retrieve other manuscripts missing in the current meta-analysis.

Drawing conclusions based on the moderate effect estimate from the meta-analysis and the certainty of the evidence according to GRADE criteria, adjuvant therapies probably increase Clostridium Difficile infections rate after stoma closure surgery, however studies analysed were observational and not RCT.

CONCLUSIONS

To conclude, the CDI is relatively low after stoma reversal surgery, and although a correlation between stoma delay closure, the use of chemotherapy and CDI population was observed, adjuvant therapy was the only significant variable showing the potential of being a possible risk factor.

Funding

The authors have no funding to declare.

Acknowledgements

The authors would like to thank Miss Cristina Vacca, Data Manager at Fondazione Policlinico Universitario 'A. Gemelli' IRCCS, for her support in data acquisition and update of the unit's database.

Disclosure

The authors declare no conflict of interest.

Data availability

The data that support the findings of this study are available on request from the corresponding author; the data are not publicly available due to privacy restrictions.

FIGURES

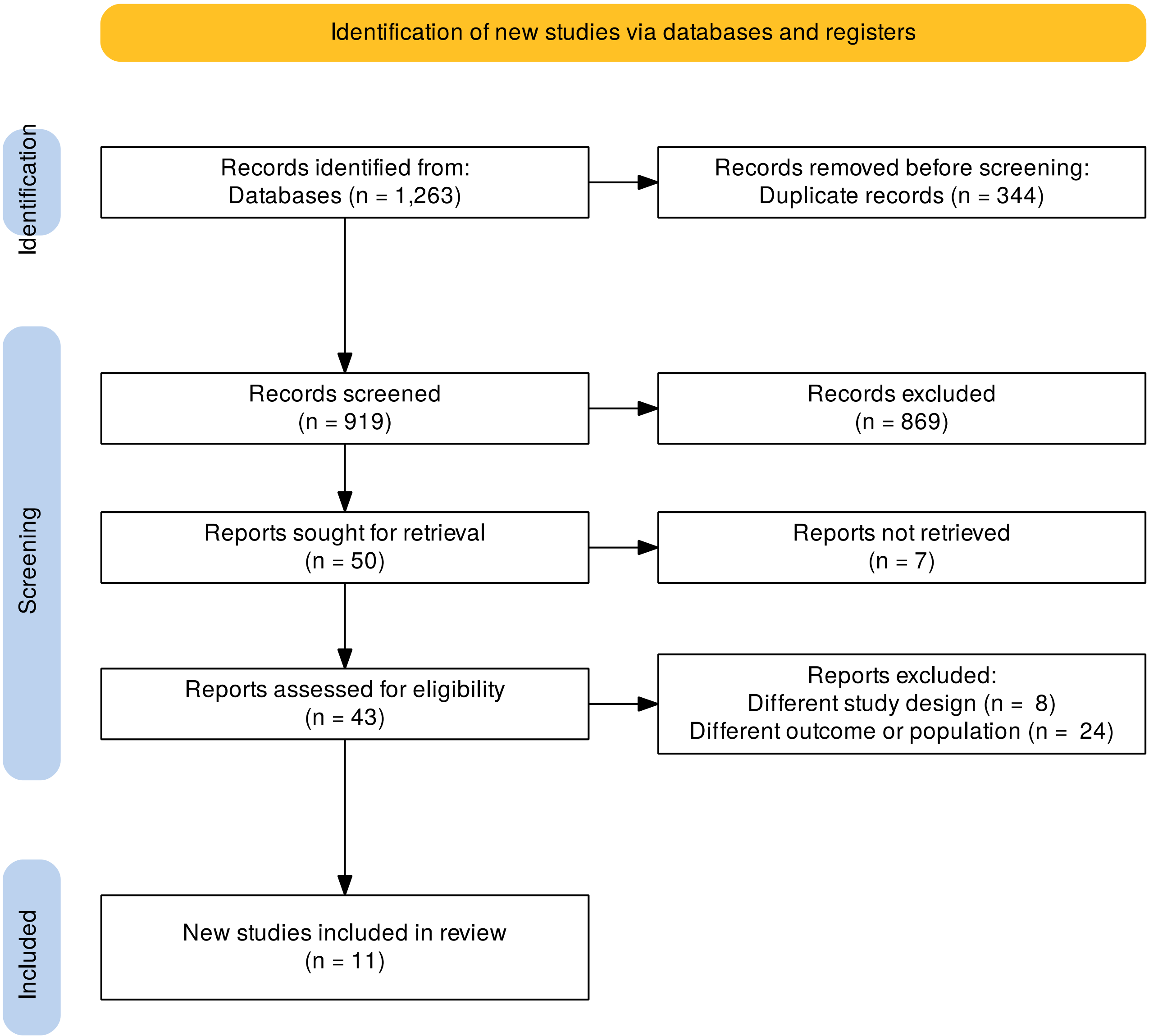

Figure 1: PRISMA 2020 flow diagram for the systematic review.
* Records identified from Ovid, Embase, and Medline using PubMed.
** Records excluded because they were not relevant.

A. Newcastle-Ottawa scale

AUTHORS	SELECTION	COMPARABILITY	OUTCOME	FINAL SCORE
**Wilson *et al.***	****	**	***	9
**Tirelli *et al.***	****	*	**	7
**Kim *et al.***	****	*	**	7
**Jordan *et al.***	****	*	**	7
**Richards *et al.***	****	*	**	7
**Skancke *et al.***	****	*	**	7
**Fernandes *et al.***	****	*	**	7
**Randall *et al.***	****	*	**	7
**Taylor *et al.***	****	*	**	7
**Lee *et al.***	****	*	**	7

B. Robin 2.0

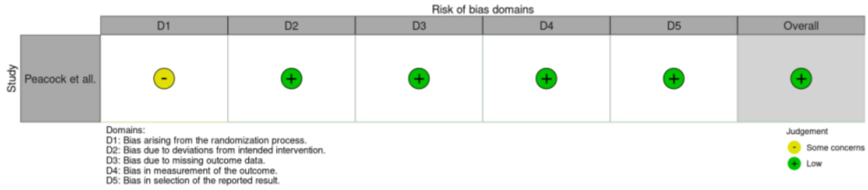

Figure 2: A. Quality assessment of the nine nonrandomized studies according to the Newcastle–Ottawa scale; **B.** Robin 2.0 for the RCTs included.

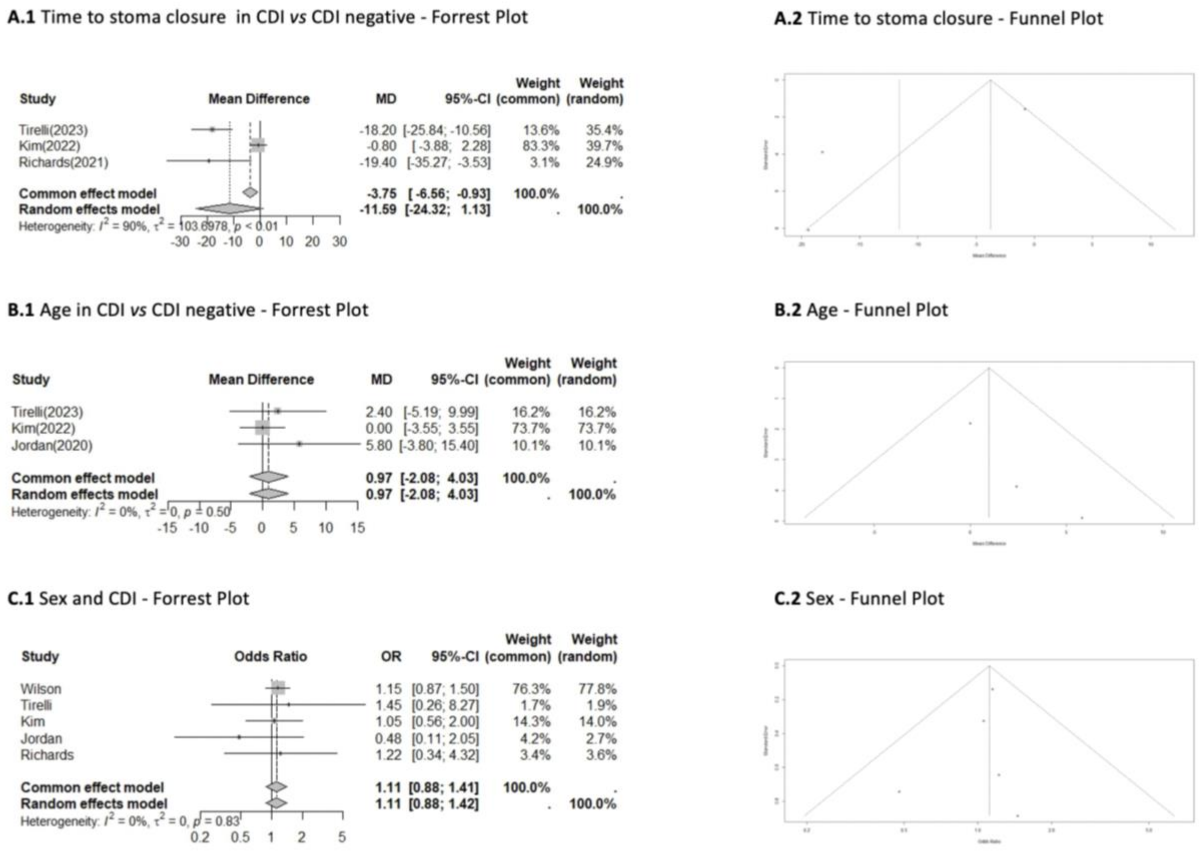

Figure 3: Meta-analysis for time to stoma closure (A1, A2), age (B1, B2) and sex (C1, C2).

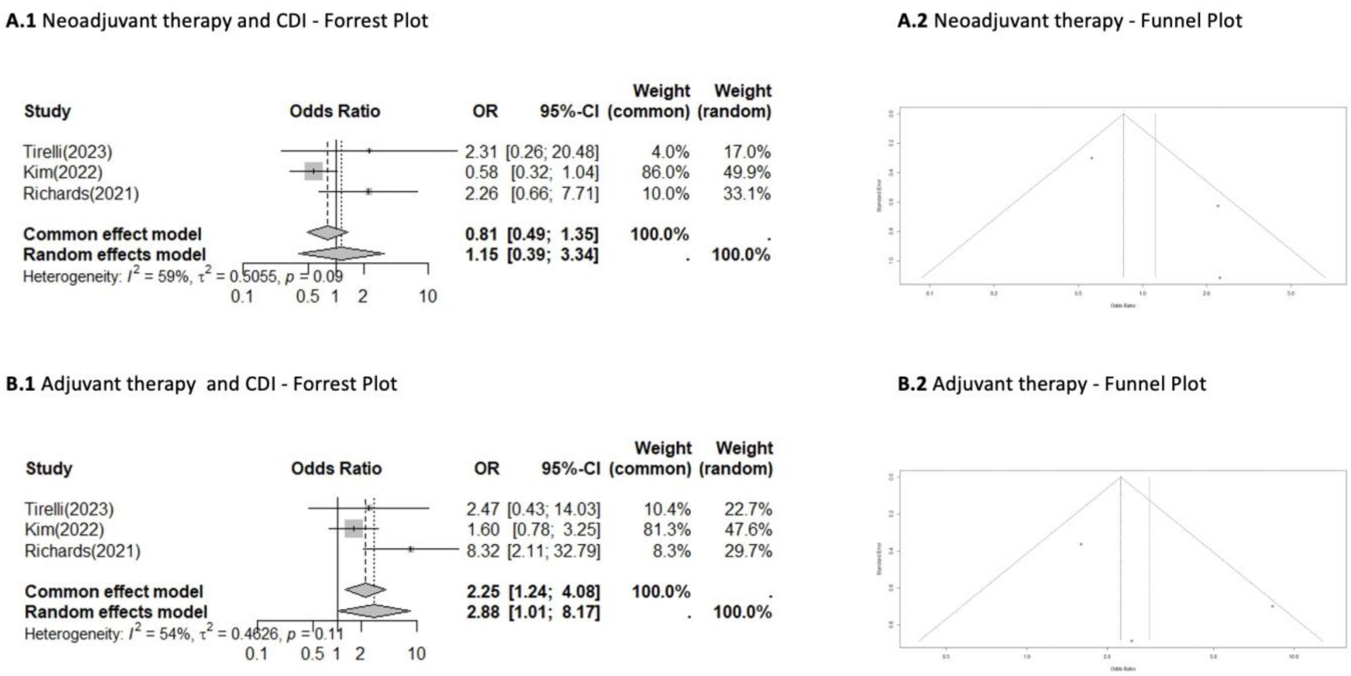

Figure 4: Meta-analysis for neo-adjuvant and adjuvant treatment.

TABLES

Table 1: Systematic review of the literature

AUTHORS	YEAR	JOURNAL	STUDY DESIGN	NUMBER OF PATIENTS	INCIDENCE OF CDI n (%)
**Wilson *et al.***	2013	Colorectal Disease	Propensity Score Matched Cohort Study	13245	217 (1.6%)
**Peacock *et al.***	2013	Tecniques of Coloproctology	Randomized Clinical Trial	23	1 (4.3%)
**Tirelli *et al.***	2023	Update in Surgery	Retrospective Cohort Study	126	6 (4.8%)
**Kim *et al.***	2022	BJS Open	Retrospective Cohort Study	1270	46 (3.6%)
**Jordan *et al.***	2020	Colorectal Surgery	Retrospective Cohort Study	228	8 (3.5%)
**Richards *et al.***	2021	Colorectal Surgery	Retrospective Cohort Study	195	11 (5.6%)
**Skancke *et al.***	2018	Disease of the colon and the rectum	Retrospective Cohort Study	2235	68 (3.0%)
**Fernandes *et al.***	2016	International Journal of Colorectal Disease	Retrospective Cohort Study	199	8 (4.0%)
**Randall *et al.***	2011	Colorectal Disease	Retrospective Cohort Study	143	6 (4.2%)
**Taylor *et al.***	2020	Royal Australasian College of Surgeon	Case Series	124	4 (3.2%)
**Lee *et al.***	2024	Cureus	Retrospective Cohort Study	69	6 (8.7%)

Table 2. Clinical variables in *Clostridium difficile* infection (CDI) patients vs controls

	CDI POSITIVE	CDI NEGATIVE	p values
**SEX (n%)**			
Female	117 (40.6%)	6727 (44.6%)	0.34*
Male	171 (59.4%)	8337 (55.3%)	
Total^	288 (100%)	15064 (100%)	
**AGE**			
Mean±SD	64.3±11.6	61.6±12.6	0.51$
Median [IQR]	64.3 [62.1–66.5]	59.8 [59.1–63.1]	
Number of Patients^^	60	1564	
**TIME TO STOMA CLOSURE**			
Mean±SD	53.9±19.1	39.8±15.0	0.40$
Median [IQR]	64.4 [45.6–66.4]	45 [35.5–47.6]	
Number of Patients^^^	63	1528	
**NEO-ADJUVANT (n%)**			
Yes	34 (11.8%)	919 (6.2%)	**<0.001***
No	254 (88.2%)	13857 (93.8%)	
Total ^^^	288 (100%)	14776 (100%)	
**ADJUVANT (n%)**			
Yes	48 (16.7%)	957 (6.5%)	**<0.001***
No	240 (83.3%)	13819 (93.5%)	
Total ^^^	288 (100%)	14776 (100%)	

Table 2:

*Chi-square test; $ Unpaired two-sample Wilcoxon test

^Results based on Wilson *et al. Colorectal Disease* 2013; Tirelli *et al. Update in Surgery* 2023; Kim *et al. BJS Open* 2022; Jordan *et al. Colorectal Surgery* 2020; Richards *et al. Colorectal Surgery* 2021

^^Results based on: Tirelli *et al Update in Surgery* 2023; Kim *et al BJS Open* 2022; Jordan *et al Colorectal Surgery* 2020

^^^ Results based on: Tirelli *et al Update in Surgery* 2023; Kim *et al BJS Open* 2022; Richards *et al Colorectal Surgery* 2021

REFERENCES

1. Sartelli M, Malangoni MA, Abu-Zidan FM *et al.* WSES guidelines for management of *Clostridium difficile* infection in surgical patients. *World J Emerg Surg* 2015; **10**: 38.

2. Yasunaga H, Horiguchi H, Hashimoto H, Matsuda S, Fushimi K. The burden of *Clostridium difficile*-associated disease following digestive tract surgery in Japan. *J Hosp Infect* 2012;**82**:175–80.

3. Yeom CH, Cho MM, Baek SK, Bae OS. Risk Factors for the Development of *Clostridium difficile*-associated Colitis after Colorectal Cancer Surgery. *J Korean Soc Coloproctol* 2010;**26**:329–33.

4. Damle RN, Cherng NB, Flahive JM, Davids JS, Maykel JA, Sturrock PR, *et al*. *Clostridium difficile* infection after colorectal surgery: a rare but costly complication. *J Gastrointest Surg* 2014;**18**:1804–11.

5. Tirelli F, Lorenzon L, Biondi A, Langellotti L, Santoro G, Agnes A, Pezzuto R, Persiani R, D'Ugo D. Predictors of *Clostridium difficile* infection after stoma reversal following TaTME surgery. *Updates Surg* 2023;**75**(6):1589-1596. doi: 10.1007/s13304-023-01614-4.

6. Wilson MZ, Hollenbeak CS, Stewart DB. Impact of *Clostridium difficile* colitis following closure of a diverting loop ileostomy: results of a matched cohort study. *Colorectal Dis* 2013;**15**(8):974-81.

7. Peacock O, Bhalla A, Simpson JA, Gold S, Hurst NG, Speake WJ, Tierney GM, Lund JN. Twenty-three-hour stay loop ileostomy closures: a pilot study. *Tech Coloproctol* 2013;**17**(1):45-9.

8. Kim YI, Yu CS, Kim YS, Kim CW, Lee JL, Yoon YS, Park IJ, Lim SB, Kim JC. *Clostridium difficile* infection after ileostomy closure and anastomotic failure in rectal cancer surgery patients. *BJS Open* 2022;**6**(2):zrac026. doi: 10.1093/bjsopen/zrac026.

9. Jordan S, Hui N, Doudle M, Von Papen M, Naik A, Lu CT, Nolan G, Cooper M. Incidence of *Clostridioides difficile* in patients post loop ileostomy reversal in an Australian tertiary hospital: a retrospective study. *ANZ J Surg* 2022;**92**(3):403-408.

10. Richards SJG, Udayasiri DK, Jones IT, Hastie IA, Chandra R, McCormick JJ, Chittleborough TJ, Read DJ, Hayes IP. Delayed ileostomy closure increases the odds of *Clostridium difficile* infection. *Colorectal Dis* 2021;**23**(12):3213-3219.

11. Skancke M, Vaziri K, Umapathi B, Amdur R, Radomski M, Obias V. Elective stoma reversal has a higher incidence of postoperative Clostridium Difficile infection compared with elective colectomy: an analysis using the American College of Surgeons National Surgical Quality Improvement Program and targeted colectomy databases. *Dis Colon Rectum* 2018;**61**(5):593-598.

12. Fernandes R, Robinson P, Rangarajan K, Scott S, Angco L. The role of single-shot metronidazole in the prevention of *Clostridium difficile* infection following ileostomy reversal surgery. *Int J Colorectal Dis* 2017;**32**(5):733-736.

13. Randall JK, Young BC, Patel G, Fitzgerald A, George BD. Is *Clostridium difficile* infection a particular problem after reversal of ileostomy? *Colorectal Dis* 2011;**13**(3):308-11.

14. Taylor D, Azher H, Gartrell R, Yeung JM. *Clostridium difficile* infection following reversal of loop ileostomy: an uncommon but significant complication. *ANZ J Surg* 2021;**91**(10):2219-2221.

15. Kissmeyer-Nielsen P, Christensen H, Laurberg S. Diverting colostomy induces mucosal and muscular atrophy in rat distal colon. *Gut* 1994; **35**:1275–1281

16. Harries RL, Ansell J, Codd RJ, Williams GL. A systematic review of *Clostridium difficile* infection following reversal of ileostomy. *Colorectal Dis* 2017;**19**(10):881-887.

17. Tirelli F, Lorenzon L, Biondi A, Neri I, Santoro G, Persiani R. Functional outcomes after transanal total mesorectal excision (TaTME): a random forest analysis to predict patients' outcomes. Tech Coloproctol. 2023 Nov;27(11):1037-1046. doi: 10.1007/s10151-023-02775-5. Epub 2023 Mar 5. PMID: 36871281; PMCID: PMC9985820.

18. Ali A, AlHussaini KI. *Helicobacter pylori*: A Contemporary Perspective on Pathogenesis, Diagnosis and Treatment Strategies. Microorganisms. 2024 Jan 22;12(1):222. doi: 10.3390/microorganisms12010222. PMID: 38276207; PMCID: PMC10818838.

19. Agnes A, La Greca A, Tirelli F, Papa V *Duodenal perforation in a SARS-CoV-2-positive patient with negative PCR results for SARS-CoV-2 in the peritoneal fluid* Eur Rev Med Pharmacol Sci 2020 Vol. 24 - N. 23 Pages: 12516-12521 DOI: 10.26355/eurrev_202012_24048

20. Lee D, Bin Abdur Raheem M, Coveney A (January 04, 2024) Single-Centre Retrospective Audit of *Clostridium difficile* Infections Post Ileostomy Reversal. Cureus 16(1): e51674. DOI 10.7759/cureus.51674
