# Supplementary material for: Clostridium difficile infection after stoma reversal surgery: a systematic review and meta-analysis of the literature
Source: Int J Colorectal Dis. 2024 May 29;39(1):81. doi: 10.1007/s00384-024-04643-6 (PMC11136761; doi:10.1007/s00384-024-04643-6)

Supplementary Figure 1. Meta-analysis of proportion for CDI Infection

A.1 Forrest Plot

Study	Proportion	95%-CI	Weight (common)	Weight (random)
Wilson	0.02	[0.01; 0.02]	74.4%	16.6%
Peacock	0.04	[0.00; 0.22]	0.1%	2.1%
Tirelli	0.05	[0.02; 0.10]	0.7%	7.4%
Kim	0.04	[0.03; 0.05]	7.1%	14.9%
Jordan	0.04	[0.02; 0.07]	1.3%	9.8%
Richards	0.06	[0.03; 0.10]	1.1%	9.2%
Skancke	0.03	[0.02; 0.04]	12.6%	15.6%
Fernandes	0.04	[0.02; 0.08]	1.1%	9.3%
Randall	0.04	[0.02; 0.09]	0.8%	7.9%
Taylor	0.03	[0.01; 0.08]	0.7%	7.3%
**Common effect model**	**0.02**	**[0.01; 0.02]**	**100.0%**	.
**Random effects model**	**0.03**	**[0.02; 0.05]**	.	**100.0%**

Heterogeneity: $I^2 = 84\%$, $\tau^2 = 0.0015$, $p < 0.01$

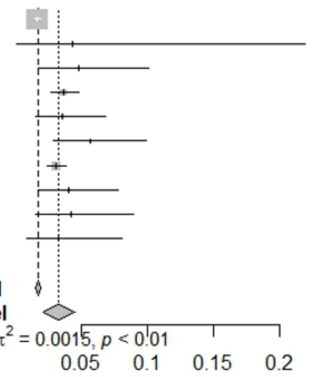

A.2 Funnel Plot

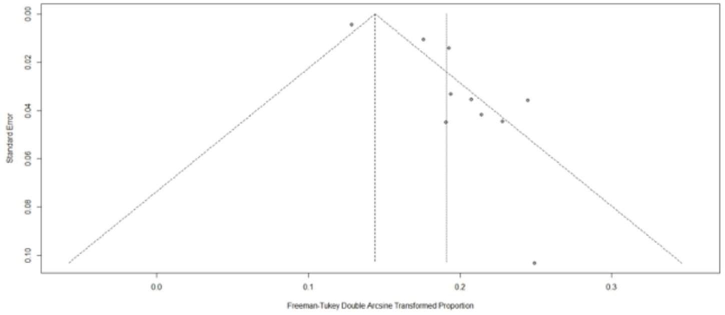

Supplement: Supplementary file 7 — Supplementary file7 (PDF 1350 kb) [file 384_2024_4643_MOESM7_ESM.pdf]
